# Recurrent and Residual Aneurysms After Woven EndoBridge (WEB) Therapy: What’s Next?

**DOI:** 10.7759/cureus.14404

**Published:** 2021-04-10

**Authors:** Catherine Peterson, Branden J Cord

**Affiliations:** 1 Neurological Surgery, University of California Davis, Sacramento, USA

**Keywords:** aneurysm, recurrence, residual, web, woven endobridge

## Abstract

The prevalence of recurrent and residual aneurysms following Woven EndoBridge (WEB) treatment is not insignificant. The goal of this systematic review was to evaluate retreatment methods for such aneurysms and their outcomes. PubMed, Embase, and Scopus databases were systematically searched, and results were reported according to the PRISMA (Preferred Reporting Items for Systematic Reviews and Meta-Analyses) guidelines. Original studies reporting on aneurysms that were retreated after WEB were included. Sixteen studies (n = 901 aneurysms), of which three were prospective, reported on retreated aneurysms following initial WEB treatment. Of those 901 aneurysms, on average 18.7 ± 11.5% were recurrent or residual at the last follow-up and 10.7 ± 11% required some form of retreatment. When compared to WEB-IT (WEB Intra-saccular Therapy) data, retreated aneurysms were more likely to be large in size (p < 0.0001) and more likely to have been initially treated with the WEB dual-layer configuration. The mean age of those with retreated aneurysms was 58 ± 5.7 years old, and the mean size of aneurysm dome was 11.1 ± 5.5 millimeters. Majority (34.1%) of the aneurysms were located at the basilar apex. Retreatment modalities included coiling (20%), stent-assisted coiling (38.7%), additional WEB device (13.3%), flow diversion (16%), and clipping (12%). Majority of retreated cases had favorable outcomes, with 96.4 ± 13.4% of the cases demonstrating technical success and 90.5 ± 18.2% having adequate occlusion at the last follow-up. Our systematic review suggests that retreatment of recurrent and residual aneurysms after initial WEB treatment is feasible. Future prospective studies would be helpful in validating these results.

## Introduction and background

The field of neuroendovascular aneurysm treatment has been rapidly evolving, with some of the most recent advancements being in the field of flow diversion. Despite the well-known techniques such as balloon and stent-assisted coiling for wide neck bifurcation intracranial aneurysms, treatment of these aneurysms has often carried its challenges. Intra-saccular flow diversion is the most recent advancement in technology for the treatment of such aneurysms. Woven EndoBridge (WEB; Sequent Medical, Aliso Viejo, CA, USA), a nitinol-based self-expanding mesh implant, is the only FDA-approved intra-saccular flow diverter device on the market today for the treatment of wide neck bifurcation aneurysms. In 2018, it was approved by the FDA to be used in adults with wide neck bifurcation aneurysms at middle cerebral artery (MCA) bifurcation, internal carotid artery terminus (ICA-T), anterior communicating artery (ACOM) complex, and basilar artery apex [1].

Despite WEB’s safety and efficacy, a significant percentage of aneurysms recanalize after initial WEB implantation [2-4]. Because of this, it is imperative to be familiar with the retreatment strategies that can be helpful in managing these aneurysms. To this day, a systematic review focusing on retreated aneurysms following initial WEB therapy has not been completed; thus, the aim of this systematic review was to explore the evidence in the current literature reporting on retreatment strategies for such aneurysms and their subsequent outcomes.

## Review

Methods

Search Strategy

The search strategy in this systematic review was conducted and reported according to the PRISMA (Preferred Reporting Items for Systematic Reviews and Meta-Analyses) guidelines [5]. The authors performed a comprehensive search of MEDLINE, Embase, and Scopus databases on August 24, 2020. Keywords or MeSH terms such as “recurrent aneurysms” OR “residual aneurysms” OR “recanalized aneurysms” OR “aneurysms” AND “WEB” or “woven endobridge” AND “coiling” OR “clipping” OR “management” OR “treatment” OR “embolization” OR “flow diversion” OR “retreatment” OR “re-treatment” were used for the search strategy.

Study Eligibility

Results were limited to original studies pertaining to humans only. Retrospective and prospective studies mentioning retreatment of intracranial aneurysms following initial WEB treatment were eligible for inclusion after the duplicates were removed. Case reports, commentaries, conference abstracts, editorials, letters, reviews, systematic reviews, meta-analyses, and non-English language studies were excluded from this systematic review. Studies without extractable or sufficient data were also excluded. For the statistical analysis portion, the retreated aneurysm cohort was compared to one of the largest prospective multicenter studies performed on WEB, the WEB Intra-saccular Therapy (WEB-IT) study [6].

Data Extraction

Data points such as demographic data, technical success of WEB implantation defined as feasibility of correctly implanting WEB device into an aneurysm without having to abort the procedure, complete or adequate (only neck remnant) endovascular aneurysmal occlusion, complications, mortality, proportion of recurrent or residual aneurysms, and proportion of retreated aneurysms were collected. The retreated aneurysm group was further assessed, and data on retreated aneurysm location, type of WEB configuration used, type of retreatment method used, and outcomes following retreatment were retrieved. A standardized computerized spreadsheet was used to collect the information from the included studies.

Statistical Analysis

For statistical analysis, the retreated aneurysm group was compared to the WEB-IT data [6]. The Student t-test was used to compare the two groups, and a p-value of < 0.05 was considered statistically significant. For statistical analysis, GraphPad Prism Version 5 was used (GraphPad Software, San Diego, CA, USA).

Results

Literature Search

**Figure 1:**
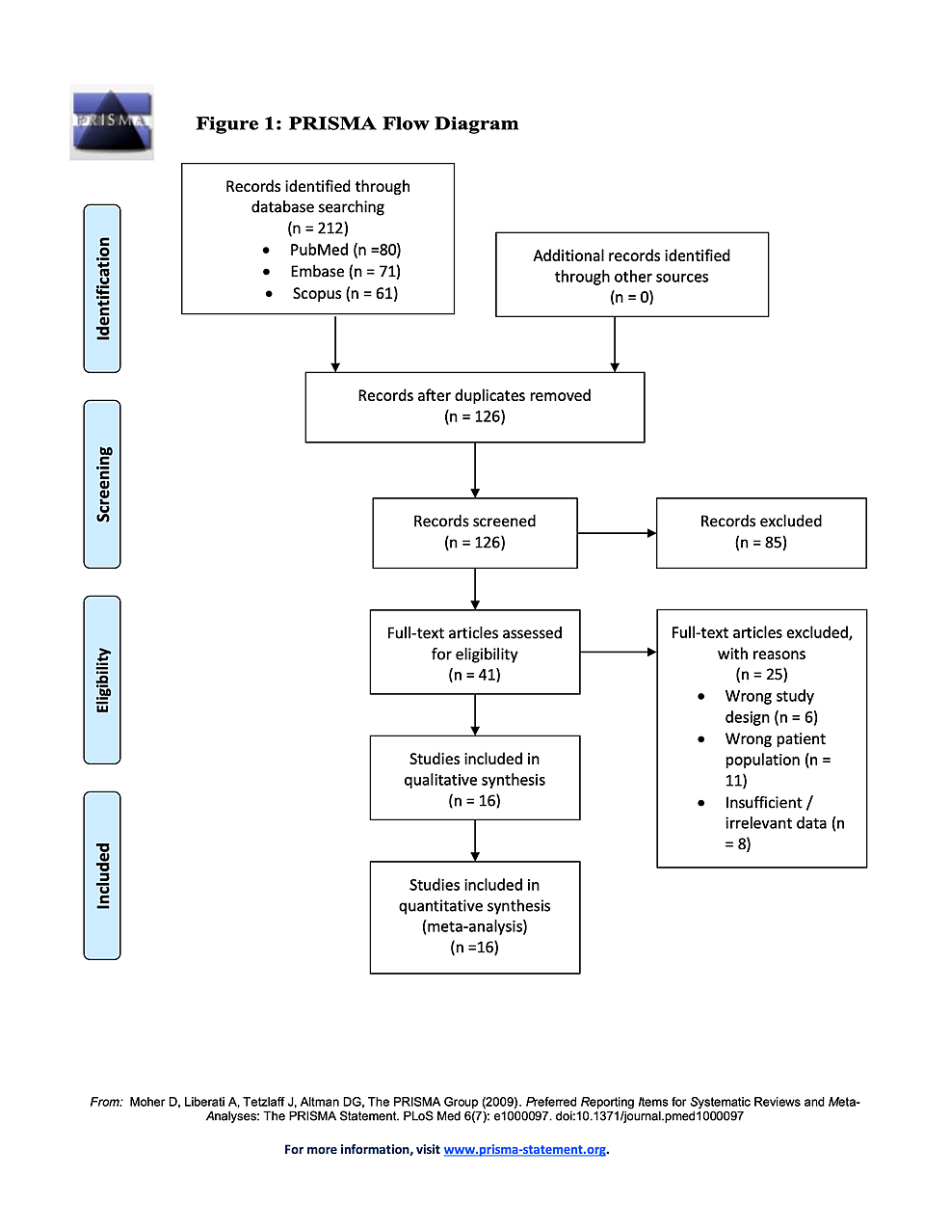
PRISMA flow diagram PRISMA, Preferred Reporting Items for Systematic Review

After designing the search strategy, a total of 212 records were identified, with 80 in PubMed, 71 in Embase, and 61 in the Scopus database (Table 1). No additional articles were identified when searching through the reference lists. After duplicates were removed and the records were initially screened by title and abstract, 41 full-text articles were then assessed for eligibility. Of those, 16 studies were found to report on retreated aneurysms after WEB therapy and those 16 studies were eligible for the systematic review (Figure 1) [7-22].

**Table 1:** Search syntax

PubMed/MEDLINE Search, Accessed on August 24, 2020 (80 Articles)	Embase Search, Accessed on August 24, 2020 (71 Articles)	Scopus Search, Accessed on August 24, 2020 (61 Articles)
(((((recurrent aneurysms) OR (residual aneurysms)) OR (recanalized aneurysms)) OR (aneurysms failed treatment)) AND ((WEB) OR (Woven EndoBridge))) AND ((((((((management) OR (treatment)) OR (embolization)) OR (retreatment)) OR (re-treatment)) OR (coiling)) OR (clipping)) OR (flow diversion))	(recurrent AND aneurysms OR (residual AND aneurysms) OR (recanalized AND aneurysms) OR (aneurysms AND failed AND treatment)) AND (web OR (woven AND endobridge)) AND (management OR treatment OR embolization OR retreatment OR 're treatment' OR coiling OR clipping OR (flow AND diversion))	((TITLE-ABS-KEY (recurrent AND aneurysms) OR TITLE-ABS-KEY (residual AND aneurysms) OR TITLE-ABS-KEY (recanalized AND aneurysms) OR TITLE-ABS-KEY (aneurysms AND failed AND treatment))) AND ((TITLE-ABS-KEY (web) OR TITLE-ABS-KEY (woven AND endobridge))) AND ((TITLE-ABS-KEY (management) OR TITLE-ABS-KEY (treatment) OR TITLE-ABS-KEY (embolization) OR TITLE-ABS-KEY (retreatment) OR TITLE-ABS-KEY (re-treatment) OR TITLE-ABS-KEY (coiling) OR TITLE-ABS-KEY (clipping) OR TITLE-ABS-KEY (flow AND diversion)))

Demographics

A total of 16 studies, of which three were prospective and the rest were retrospective in design, had a total of 901 WEB cases performed and reported on retreatment following initial WEB therapy (Table 2). Of those 16 studies, the mean age was 57.8 ± 2.4 years old, and 71.1 ± 7.3% were female. The mean technical success rate of WEB placement was high and was achieved in 96.5 ± 3.9% of the cases. Mean adequate aneurysm occlusion was observed in 83 ± 9.6% of the cases at the last follow-up. Mean mortality was seen in 3.2 ± 4.6% of the cases, and mean thromboembolic complication rate was 9.2 ± 4%. There were 18.7 ± 11.5% cases of either recurrent or residual aneurysms following initial WEB therapy and 10.7 ± 11% had to undergo retreatment of some sort.

**Table 2:** Characteristics and demographics of included studies Values are reported as means ± standard deviation unless otherwise specified. WEB, Woven EndoBridge

Characteristics and demographics	
Studies included in the systematic review	16
Total number of WEB cases	901
Female sex	71.1 ± 7.3%
Mean age (years)	57.8 ± 2.4
Technical success of WEB	96.5 ± 3.9%
Complete aneurysm occlusion at the last follow-up	51.7 ± 18.6%
Adequate aneurysm occlusion at the last follow-up	83 ± 9.6%
Mortality	3.2 ± 4.6%
Thromboembolic complications	9.2 ± 4%
Recurrent or residual aneurysms after WEB	18.7 ± 11.5%
Retreated aneurysms after WEB	10.7 ± 11%

Retreated Aneurysms

**Figure 2:**
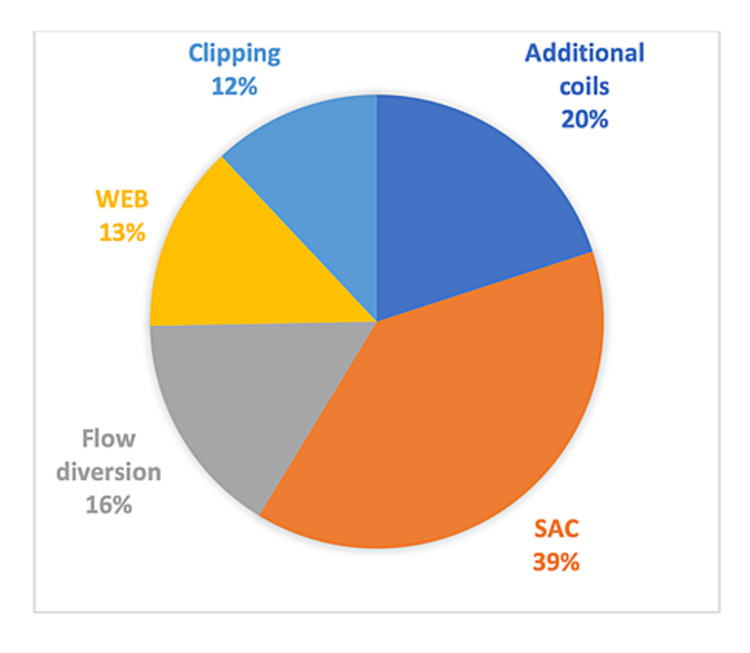
Retreatment strategies Pie diagram of retreatment strategies used for recurrent or residual aneurysms after initial WEB therapy SAC, stent-assisted coiling; WEB, Woven EndoBridge

The retreated aneurysm cohort was further analyzed (Table 3). Mean age of retreated aneurysms following initial WEB implantation was 58 ± 5.7 years. The mean size of dome width was 11.1 ± 5.5 millimeters. Approximately, a quarter of those aneurysms were ruptured initially. Majority (34.1%) of the aneurysms were located at the basilar apex followed by 31.8% at ACOM, 18.2% at MCA bifurcation, and 15.9% at the ICA-T. Approximately half of these aneurysms were initially treated with the WEB dual-layer (WEB-DL) device. Various retreatment strategies were utilized, with the majority being stent-assisted coiling (Figure 2). The retreatment was technically successful in mean 96.4 ± 13.4% of the cases, with 90.5 ± 18.2% of the retreated aneurysms achieving adequate occlusion at the last follow-up. Mean complication and mortality rates following retreatment were low, with 2.6 ± 7.7% and 1 ± 3.7% of the cases, respectively. When the retreated aneurysm group was compared to the WEB-IT study results, it was found that a significantly higher proportion of retreated aneurysms (47.8%) were initially treated with WEB-DL device, whereas only 12.8% of the WEB cases were treated with the WEB-DL configuration in the WEB-IT prospective trial (Table 4). The mean age between the two groups was similar (p = 0.636); however, the retreated aneurysms were significantly larger in size compared to the average aneurysm size from the WEB-IT study data (p < 0.0001).

**Table 3:** Retreated aneurysms following initial WEB treatment Values are reported as means ± standard deviation unless otherwise specified. ACOM, anterior communicating artery; ICA-T, internal carotid artery terminus; MCA, middle cerebral artery; SAC, stent-assisted coiling; WEB-DL, Woven EndoBridge dual layer; WEB-SL/SLS, Woven EndoBridge single layer/single layer sphere

Characteristics of retreated aneurysms following initial WEB treatment	
Retreated aneurysms after WEB	10.7 ± 11%
Mean age (years)	58 ± 5.7
Mean size of dome (mm)	11.1 ± 5.5
Ruptured retreated aneurysms	25.4 ± 32.6%
Location of retreated aneurysms	-
ICA-T	15.9%
MCA	18.2%
Basilar	34.1%
ACOM	31.8%
Initial WEB configuration used	-
WEB-DL	47.8%
WEB-SL/SLS	52.2%
Types of retreatment	-
Additional coils	20%
SAC	38.7%
Flow diversion	16%
WEB	13.3%
Clipping	12%
Technical success of retreatment	96.4 ± 13.4%
Adequate occlusion after retreatment	90.5 ± 18.2%
Complications of retreatment	2.6 ± 7.7%
Mortality after retreatment	1 ± 3.7%

**Table 4:** Retreated aneurysm cohort versus WEB-IT study Values are reported as means ± standard deviation unless otherwise specified. A p-value of < 0.05 was considered statistically significant. WEB-DL, Woven EndoBridge dual layer; WEB-IT, Woven EndoBridge Intra-saccular Therapy; WEB-SL/SLS, Woven EndoBridge single layer/single layer sphere

	Retreated aneurysms	WEB-IT study	p-Value
Mean age (years)	58 ± 5.7	59 ± 12.5	0.636
Mean size of dome (mm)	11.1 ± 5.5	6.4 ± 1.95	<0.0001
Ruptured	25.4%	6%	-
Anterior circulation	65.9%	60.7%	-
Posterior circulation	34.1%	39.3%	-
Initial WEB-DL	47.8%	12.8%	-
Initial WEB-SL/SLS	52.2%	87.2%	-

Discussion 

This is the first systematic review that focuses mainly on the retreatment of recurrent and residual aneurysms after initial WEB therapy. Our results suggest that a significant proportion of aneurysms following WEB implantation have to be retreated. Retreatment modalities vary, with stent-assisted coiling being the most common retreatment strategy reported in the current literature. Despite this, retreatment of recurrent and residual aneurysms is technically feasible, with an overall good occlusion rate and low complication rate. Furthermore, our data demonstrate that aneurysms requiring retreatment are more likely to be large in size and to have been initially treated with the WEB-DL configuration.

Alternative techniques such as WEB for treating wide neck aneurysms are being used more often. WEB is a nitinol-based electrothermally detachable device that functions as an intra-saccular flow diverter. It is currently approved by the FDA for the treatment of wide neck bifurcation intracranial aneurysms. It works by disrupting the blood flow across the neck of the aneurysm, thus promoting its thrombosis. It does not rely on placement of an intravascular device, eliminating the need for long-term dual antiplatelet therapy. In the early stages, first-generation WEB was a WEB-DL device, which was a dual-layered device with the second nitinol cage placed inside the first nitinol braid. Later, newer generation of WEB devices, such as single-layer configurations (WEB-SL/SLS), replaced the WEB DL. WEB comes in various sizes, but the recommended aneurysm size treatment range is 2-10 millimeters [1].

There is a plethora of evidence in the current literature demonstrating WEB’s effectiveness and safety for the treatment of wide neck bifurcation aneurysms [23-30]. Most recent data demonstrate that the overall rate of adequate aneurysm occlusion at the last follow-up after WEB therapy is approximately 80% [2-3]. Nonetheless, approximately 10% of all aneurysms treated with WEB will recanalize. Zhang et al. found variables such as ruptured status, posterior circulation, and old-generation WEB to be associated with higher recanalization rates [3].

A recent systematic review by van Rooij et al. found only eight studies that reported on retreatment after WEB, with mean retreatment rate of 8.4% following initial WEB implantation; however, that study did not analyze the retreated cohort of aneurysms in detail [2]. Indeed, the results reported in our study reflect a similar rate of retreatment following WEB. Our systematic review focused only on studies that reported retreatment after WEB, and we were able to further analyze the retreated aneurysms. We found that a high percentage of them were initially treated with the first-generation WEB-DL device. As WEB-SL/SLS devices are used more often in the most recent years, and with the newer generations being developed in the near future, we can suspect that the rate of retreatment following initial WEB therapy should decrease. Dome size was another variable we found herein to be associated with aneurysms that required retreatment. We found the mean size of retreated aneurysms to be 11.1 ± 5.5 millimeters, disproportionally larger than what is currently recommended for WEB implantation [1].

## Conclusions

In conclusion, this is the first systematic review that focused on recurrent and residual aneurysms that required retreatment following initial WEB implantation. Our results suggest that intracranial wide neck bifurcation aneurysms requiring retreatment are more likely to be large in size and to have been initially treated with the older WEB-DL configuration. Retreatment modalities for such aneurysms greatly vary but carry high technical success rate and low complication rates in the literature. Sufficient knowledge of proper indications for the use of WEB and perhaps the development of newer generations and configurations of the WEB device in the near future may aid in avoiding the need for retreatment.

## References

[1] Gawlitza M, Soize S, Manceau PF, Pierot L (2019). An update on intrasaccular flow disruption for the treatment of intracranial aneurysms. Expert Rev Med Devices.

[2] van Rooij S, Sprengers ME, Peluso JP, Daams J, Verbaan D, van Rooij WJ, Majoie CB (2020). A systematic review and meta-analysis of Woven EndoBridge single layer for treatment of intracranial aneurysms. Interv Neuroradiol.

[3] Zhang SM, Liu LX, Ren PW, Xie XD, Miao J (2020). Effectiveness, safety and risk factors of Woven EndoBridge device in the treatment of wide-neck intracranial aneurysms: systematic review and meta-analysis. World Neurosurg.

[4] Asnafi S, Rouchaud A, Pierot L, Brinjikji W, Murad MH, Kallmes DF (2016). Efficacy and safety of the Woven EndoBridge (WEB) device for the treatment of intracranial aneurysms: a systematic review and meta-analysis. AJNR Am J Neuroradiol.

[5] Moher D, Liberati A, Tetzlaff J, Altman DG (2009). Preferred reporting items for systematic reviews and meta-analyses: the PRISMA statement. PLoS Med.

[6] Fiorella D, Molyneux A, Coon A (2017). Demographic, procedural and 30-day safety results from the WEB Intra-saccular Therapy Study (WEB-IT). J Neurointerv Surg.

[7] Arthur AS, Molyneux A, Coon AL (2019). The safety and effectiveness of the Woven EndoBridge (WEB) system for the treatment of wide-necked bifurcation aneurysms: final 12-month results of the pivotal WEB Intrasaccular Therapy (WEB-IT) Study. J Neurointerv Surg.

[8] Clajus C, Strasilla C, Fiebig T, Sychra V, Fiorella D, Klisch J (2017). Initial and mid-term results from 108 consecutive patients with cerebral aneurysms treated with the WEB device. J Neurointerv Surg.

[9] Lubicz B, Mine B, Collignon L, Brisbois D, Duckwiler G, Strother C (2013). WEB device for endovascular treatment of wide-neck bifurcation aneurysms. AJNR Am J Neuroradiol.

[10] Goertz L, Liebig T, Siebert E (2019). Extending the indication of Woven EndoBridge (WEB) embolization to internal carotid artery aneurysms: a multicenter safety and feasibility study. World Neurosurg.

[11] Kabbasch C, Goertz L, Siebert E, Herzberg M, Borggrefe J, Dorn F, Liebig T (2019). Factors that determine aneurysm occlusion after embolization with the Woven EndoBridge (WEB). J Neurointerv Surg.

[12] Khalid Z, Sorteberg W, Nedregaard B, Sorteberg A (2019). Efficiency and complications of Woven EndoBridge (WEB) devices for treatment of larger, complex intracranial aneurysms-a single-center experience. Acta Neurochir (Wien).

[13] Lawson A, Goddard T, Ross S, Tyagi A, Deniz K, Patankar T (2017). Endovascular treatment of cerebral aneurysms using the Woven EndoBridge technique in a single center: preliminary results. J Neurosurg.

[14] Lescher S, du Mesnil de Rochemont R, Berkefeld J (2016). Woven Endobridge (WEB) device for endovascular treatment of complex unruptured aneurysms-a single center experience. Neuroradiology.

[15] Liebig T, Kabbasch C, Strasilla C (2015). Intrasaccular flow disruption in acutely ruptured aneurysms: a multicenter retrospective review of the use of the WEB. AJNR Am J Neuroradiol.

[16] Ozpeynirci Y, Braun M, Pala A, Schick M, Schmitz B (2019). WEB-only treatment of ruptured and unruptured intracranial aneurysms: a retrospective analysis of 47 aneurysms. Acta Neurochir (Wien).

[17] Pierot L, Liebig T, Sychra V (2012). Intrasaccular flow-disruption treatment of intracranial aneurysms: preliminary results of a multicenter clinical study. AJNR Am J Neuroradiol.

[18] Pierot L, Moret J, Turjman F (2016). WEB treatment of intracranial aneurysms: clinical and anatomic results in the french observatory. AJNR Am J Neuroradiol.

[19] van Rooij S, van Rooij WJ, Sluzewski M, Peluso JP (2019). The Woven EndoBridge (WEB) for recurrent aneurysms: clinical and imaging results. Interv Neuroradiol.

[20] Youssef PP, Dornbos III D, Peterson J (2020). Woven EndoBridge (WEB) device in the treatment of ruptured aneurysms [Online ahead of print]. J Neurointerv Surg.

[21] Lubicz B, Klisch J, Gauvrit JY (2014). WEB-DL endovascular treatment of wide-neck bifurcation aneurysms: short- and midterm results in a European study. AJNR Am J Neuroradiol.

[22] Pierot L, Bannery C, Batchinsky-Parrou V, Kleiber JC, Soize S, Litre CF (2019). Clipping of recanalized intracerebral aneurysms initially treated by the Woven EndoBridge device. J Neurointerv Surg.

[23] Ding YH, Lewis DA, Kadirvel R, Dai D, Kallmes DF (2011). The Woven EndoBridge: a new aneurysm occlusion device. AJNR Am J Neuroradiol.

[24] Fujimoto M, Lylyk I, Bleise C, Albiña P, Chudyk J, Lylyk P (2020). Long-term outcomes of the WEB device for treatment of wide-neck bifurcation aneurysms. AJNR Am J Neuroradiol.

[25] Pierot L, Moret J, Barreau X (2020). Aneurysm treatment with Woven EndoBridge in the cumulative population of 3 prospective, multicenter series: 2-year follow-up. Neurosurgery.

[26] Cagnazzo F, Ahmed R, Zannoni R (2019). Predicting factors of angiographic aneurysm occlusion after treatment with the Woven EndoBridge device: a single-center experience with midterm follow-up. AJNR Am J Neuroradiol.

[27] Da Ros V, Bozzi A, Comelli C (2019). Ruptured intracranial aneurysms treated with Woven EndoBridge intrasaccular flow disruptor: a multicenter experience. World Neurosurg.

[28] Kabbasch C, Goertz L, Siebert E (2019). WEB embolization versus stent-assisted coiling: comparison of complication rates and angiographic outcomes. J Neurointerv Surg.

[29] Nguyen HA, Soize S, Manceau PF, Vudang L, Pierot L (2020). Persistent blood flow inside the Woven EndoBridge device more than 6 months after intracranial aneurysm treatment: frequency, mechanisms, and management—a retrospective single-center study. AJNR Am J Neuroradiol.

[30] Raj R, Rautio R, Pekkola J, Rahi M, Sillanpää M, Numminen J (2019). Treatment of ruptured intracranial aneurysms using the Woven EndoBridge device: a two-center experience. World Neurosurg.

